# Diagnostic and Screening AI Tools in Brazil’s Resource-Limited Settings: Systematic Review

**DOI:** 10.2196/69547

**Published:** 2025-09-10

**Authors:** Leticia Medeiros Mancini, Luiz Eduardo Vanderlei Torres, Jorge Artur P de M Coelho, Nichollas Botelho da Fonseca, Pedro Fellipe Dantas Cordeiro, Samara Silva Noronha Cavalcante, Diego Dermeval

**Affiliations:** 1 Faculty of Medicine, Universidade Federal de Alagoas, Av. Lourival Melo Mota, S/n - Tabuleiro do MartinsMaceió, 57072-900, Brazil, 558232141461

**Keywords:** artificial intelligence, Brazil, diagnosis, screening, PRISMA

## Abstract

**Background:**

Artificial intelligence (AI) has the potential to transform global health care, with extensive application in Brazil, particularly for diagnosis and screening.

**Objective:**

This study aimed to conduct a systematic review to understand AI applications in Brazilian health care, especially focusing on the resource-constrained environments.

**Methods:**

A systematic review was performed. The search strategy included the following databases: PubMed, Cochrane Library, Embase, Web of Science, LILACS, and SciELO. The search covered papers from 1993 to November 2023, with an initial overview of 714 papers found, of which 25 papers were selected for the final sample. Meta-analysis data were evaluated based on three main metrics: area under the receiver operating characteristic curve, sensitivity, and specificity. A random effects model was applied for each metric to address study variability.

**Results:**

Key specialties for AI tools include ophthalmology and infectious disease, with a significant concentration of studies conducted in São Paulo state (13/25, 52%). All papers included testing to evaluate and validate the tools; however, only two conducted secondary testing with a different population. In terms of risk of bias, 10 of 25 (40%) papers had medium risk, 8 of 25 (32%) had low risk, and 7 of 25 (28%) had high risk. Most studies were public initiatives, totaling 17 of 25 (68%), while 5 of 25 (20%) were private. In limited-income countries like Brazil, minimum technological requirements for implementing AI in health care must be carefully considered due to financial limitations and often insufficient technological infrastructure. Of the papers reviewed, 19 of 25 (76%) used computers, and 18 of 25 (72%) required the Windows operating system. The most used AI algorithm was machine learning (11/25, 44%). The combined sensitivity was 0.8113, the combined specificity was 0.7417, and the combined area under the receiver operating characteristic curve was 0.8308, all with *P*<.001.

**Conclusions:**

There is a relative balance in the use of both diagnostic and screening tools, with widespread application across Brazil in varied contexts. The need for secondary testing highlights opportunities for future research.

## Introduction

Artificial intelligence (AI) can be defined in various ways, often simplified as an “imitation” of the human mind. However, these programs go beyond this definition, as they operate with different datasets and levels of autonomy, refined according to the goals set by developers. Given these technologies’ vast applications, a significant transformation is occurring in the global health landscape. AI demonstrates utility in several areas, with some of its main competencies being differential diagnoses, lesion identification in imaging exams, and mortality prediction in hospitals [1-3].

In this context, it is crucial to recognize that the AI model developed is a significant element in the process, but the application environment is equally important. Using these tools in different environments depends on the resources available in each. Thus, it is necessary to consider how inequality in device access and the particularities of each location may interfere with the results, accuracy, and safety of the technologies used [4].

Various models have been applied in Brazil, emphasizing diagnostic and screening areas. Screening is used to identify individuals at risk of a given condition, prioritizing sensitivity maximization. In contrast, diagnosis aims to confirm or rule out the presence of a condition in individuals already identified as at risk. In these cases, accuracy becomes crucial, requiring a more careful balance between sensitivity and specificity. Sensitivity refers to the percentage of positive results among individuals with a given disease or clinical condition. At the same time, specificity denotes the test’s ability to yield negative results in individuals who do not have the disease under investigation. These tools are developed by the public and private sectors, despite 70% of the Brazilian population relying on the Unified Health System (UHS), the public health care system used in the country. It spans the entire territory and all levels of health care, ensuring comprehensive, universal, and free access for the whole population. Understanding how these technologies are used in sectors with more abundant resources can serve as a foundation for future implementations in UHS [5].

Therefore, this study aims to conduct a systematic literature review on the application of AI in health in Brazil, focusing on addressing the following questions: Is the tool used in the research for diagnosis or screening? What is the context and location of the tool’s application? Is the initiative public or private? Was the research funded? If so, by whom? What is the area or specialty of the tool? What type of AI app is used? What are the minimum requirements for using the tool? Was the tool tested? Was the tool tested on a population different from the one used to create the device? Was there evidence of health improvement?

These questions are fundamental for evaluating the effectiveness, accessibility, and safety of AI technologies in the Brazilian context, as detailed in Textbox 1. Thus, the review aims to understand the use of AI in the health area in Brazil, with emphasis on scenarios of limited resources, enabling the understanding of how these technologies are applied in the Brazilian reality, in order to visualize possible improvements to public health for a large part of this population.

Textbox 1. **Research questions and motivation.**

**RQ1: Is the tool used for diagnosis or screening?**

- This question helps clarify the primary objective of each intervention. This distinction is essential, as screening focuses on maximizing sensitivity to ensure few actual cases are missed, while diagnosis aims to confirm or rule out a condition in individuals already identified as at risk, requiring a balance between sensitivity and specificity.

**RQ2: What is the context and location of the tool’s application?**

- The context of application is crucial to understanding the tool’s target audience, as well as where and how it would be used. Additionally, the location indicates where these tools are more widely adopted and developed, highlighting the main centers advancing these technologies.

**RQ3: Is the initiative public or private?**

- This question helps indicate whether the tool’s purpose leans more toward improving the public health sector in the case of public initiatives or if private initiatives might focus more on cutting-edge technological innovation, operational efficiency, and potentially commercializing tools.

**RQ4: Was the research funded? If so, by whom?**

- This question may influence the development, testing, implementation, and materials used for the tool.

**RQ5: What is the field or specialty of the tool?**

- The tool’s field is essential to understand the specific needs of that specialty, ranging from data requirements to expected outcomes.

**RQ6: What type of artificial intelligence (AI) application is it?**

- This question clarifies the technological approach adopted and its implications for the tool’s performance. Different types of AI have distinct capabilities and limitations that can directly impact the tool’s accuracy, adaptability, and complexity. This choice affects model robustness, data types analyzed, and consequently, tool effectiveness.

**RQ7: What are the minimum requirements for using the tool?**

- This question is essential for assessing implementation feasibility in limited-income contexts like Brazil, as financial and technological constraints are crucial to ensuring effective and accessible tool use.

**RQ8: Was the tool tested?**

- This question is critical, as tool testing is necessary to validate its efficacy, accuracy, and safety before clinical implementation.

**RQ9: Was the tool tested on a population different from the one used to create the device?**

- This question assesses the tool’s applicability across different contexts and populations, helping avoid sample bias and ensure functionality across diverse settings.

## Methods

### Search Strategy and Selection Process

The search strategy was conducted in the following databases: PubMed, Cochrane Library, Embase, Web of Science, LILACS, and SciELO. Multimedia Appendix 1 presents the search strategies adapted for each database. The Rayyan app was used for duplicate study removal, and 5 different authors independently used it with the blind mode enabled to assess study selection [6]. The first phase involved analyzing the title, abstract, and keywords; at the end of this phase, if there was a disagreement, the authors met to discuss the application of inclusion and exclusion criteria for each study. The second phase involved a full-text reading. The searches identified papers from 1993 to November 2023, leaving 25 papers for the final sample. This systematic review and meta-analysis followed the PRISMA (Preferred Reporting Items for Systematic Reviews and Meta-Analyses) guidelines.

### Inclusion and Exclusion Criteria

The inclusion criteria for this study consisted of papers in which AI is applied within the health sectors in Brazil as an aid for diagnosis and screening of pathologies, restricted to publications in English and Portuguese. Conversely, exclusion criteria were established to eliminate duplicate papers, literature reviews, animal studies, and studies where AI does not apply in the Brazilian context. Papers exclusively focused on robotic surgeries, AI using only within scientific methodology, hospital resource management, test reading, risk stratification and prognosis, risk factor assessment, epidemiological surveillance strategies, or any other topic unrelated to diagnosis and screening of pathologies were excluded.

### Data Extraction

Data extracted from each study included: authors and publication date, location and application context, purpose (diagnosis or screening), type of initiative (public or private), study funding, benefiting specialty, type of AI application, whether tested in the population, tool functionality, and validation method. Additionally, values for accuracy, recall, precision, sensitivity, specificity, positive predictive value, and negative predictive value were obtained. Finally, the risk of bias was analyzed using the PROBAST (Prediction model Risk of Bias Assessment Tool; Table 1) to complete a risk assessment table based on the study data [7].

**Table 1.** Risk of bias questions.

	Question	Possible answers
QV1	Were appropriate data sources used, for example, cohort, randomized controlled trial, or nested case-control study data?	Y/N ?
QV2	Were all the inclusions and exclusions of participants appropriate?	Y/N ?
QV3	Were predictors defined and assessed in a similar way for all participants?	Y/N ?
QV4	Were predictor assessments made without knowledge of outcome data?	Y/N ?
QV5	Were all predictors available at the time the model was intended to be used?	Y/N ?
QV6	Was the outcome determined appropriately?	Y N ?
QV7	Was a prespecified or standard outcome definition used?	Y/N ?
QV8	Were predictors excluded from the outcome definition?	Y/N ?
QV9	Was the outcome defined and determined in a similar way for all participants?	Y/N ?
QV10	Was the outcome determined without knowledge of predictor information?	Y/N ?
QV11	Was the time interval between predictor assessment and outcome determination?	Y/N ?
QV12	Were there a reasonable number of participants with the outcome?	Y/N ?
QV13	Were continuous and categorical predictors handled appropriately?	Y/N ?
QV14	Were all enrolled participants included in the analysis?	Y/N ?
QV15	Were participants with missing data handled appropriately?	Y/N ?
QV16	Was the selection of predictors based on univariable analysis avoided?	Y/N ?
QV17	Were complexities in the data (eg, censoring, competing risks, and sampling of control participants) accounted for appropriately?	Y/N ?
QV18	Were relevant model performance measures evaluated appropriately?	Y/N ?
QV19	Were model overfitting and optimism in model performance accounted for?	Y/N ?

### Statistical Analysis

Quantitative methods were adopted to perform the statistical analysis of the studies included in this systematic review, aiming to synthesize and interpret the collected data. First, a descriptive analysis was conducted on study characteristics, such as type of initiative (public or private), application areas, and type of AI used (diagnosis or screening). These data were categorized and presented in absolute and relative frequencies.

Additionally, a quantitative analysis was conducted to assess the distribution of funding, classifying it as public, private, or mixed. The proportion of tested versus untested tools was evaluated to understand the robustness of the evidence presented by the studies. For issues related to application context and location, frequencies and percentages were calculated for different regions in Brazil. Finally, the analysis explored whether there was evidence of health improvement in studies that tested their tools.

Meta-analysis data were evaluated based on three main metrics: area under the receiver operating characteristic curve (AUC), sensitivity, and specificity. A random effects model was used for each metric to address the variability among the included studies. The exclusion of missing data was essential to ensure the quality and integrity of the results obtained. The AUC is used to measure a diagnostic test’s performance. The AUC ranges from 0.5 to 1.0: an area of 0.5 suggests that the diagnostic test has no discriminatory ability, while an area of 1.0 is considered the ideal test with perfect diagnostic accuracy [8].

The primary analysis used a random-effects model to encompass heterogeneity across studies. Cochran Q statistic was used to evaluate overall heterogeneity, and the *I*² statistic was calculated to quantify the level of heterogeneity.

All statistical analyses were performed using R software(version 4.4.2, R Foundation for Statistical Computing) and RStudio (version 2024.09.1+394, RStudio, PBC), and the statistical significance level was set at α=0.05.

## Results

### Overview

**Figure 1.**
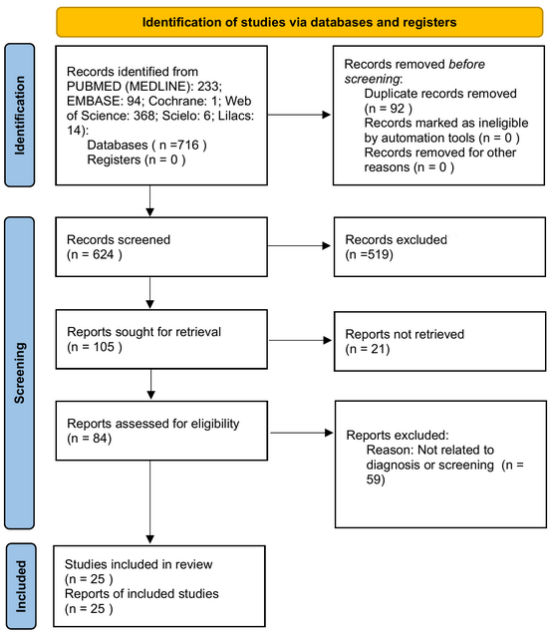
PRISMA study selection flowchart. PRISMA: Preferred Reporting Items for Systematic Reviews and Meta-Analyses.

The study was funded by the National Council for Scientific and Technological Development (CNPq), and the data analysis has already been completed, with a meta-analysis using a random effects model. After searching the databases, 714 papers were found, of which 233 were from the PubMed database, 94 from Embase, 1 from Cochrane, 368 from Web of Science, 6 from SciELO, and 14 from Lilacs. After removing duplicates, 624 studies remained. In the first selection phase, applying the inclusion and exclusion criteria by analyzing the title, abstract, and keywords, 105 papers were selected for full-text reading. However, after removing inaccessible, incomplete papers or those not meeting the previously defined exclusion criteria, 84 papers remained. Subsequently, papers that did not involve the diagnostic and screening process were removed, resulting in a final sample of 25 studies for this review, as illustrated in Figure 1. The papers are identified in Table 2.

**Table 2.** Identification.

Author (year)	ID	Study title
de Araújo et al (2020) [9]	8	DZC DIAG: mobile application based on expert system to aid in the diagnosis of dengue, Zika, and chikungunya
Shigueoka et al (2018) [8]	9	Automated algorithms combining structure and function outperform general ophthalmologists in diagnosing glaucoma
Tenório et al (2011) [10]	10	Artificial intelligence techniques applied to the development of a decision-support system for diagnosing celiac disease
Brito et al (2021) [11]	11	Left ventricular systolic dysfunction predicted by artificial intelligence using the electrocardiogram in Chagas disease patients—The SaMi-Trop cohort.
Filho et al (2018) [12]	12	Screening for active pulmonary tuberculosis: development and applicability of artificial neural network models
Malerbi et al (2022) [13]	13	Diabetic retinopathy screening using artificial intelligence and handheld smartphone-based retinal camera
Albuquerque et al (2023) [14]	14	Osteoporosis screening using machine learning and electromagnetic waves
Tavares et al (2022) [15]	15	Prediction of metabolic syndrome: a machine learning approach to help primary prevention
de Oliveira et al (2023) [16]	16	Clinical validation of a smartphone-based retinal camera for diabetic retinopathy screening
Jidling et al (2023) [17]	17	Screening for Chagas disease from the electrocardiogram using a deep neural network
Filho et al (2021) [18]	18	Can machine learning be useful as a screening tool for depression in primary care?
Giavina-Bianchi et al (2021) [19]	19	Implementation of artificial intelligence algorithms for melanoma screening in a primary care setting
Fleury et al (2020) [20]	20	Impact of radiomics on the breast ultrasound radiologist’s clinical practice: from lumpologist to data wrangler
Miranda and Felipe (2015) [21]	21	Computer-aided diagnosis system based on fuzzy logic for breast cancer categorization
Takao et al (2022) [22]	22	Artificial intelligence in allergy and immunology: comparing risk prediction models to help screen inborn errors of immunity
Filho et al (2023) [23]	23	An online platform for COVID-19 diagnostic screening using a machine learning algorithm
Delafiori et al (2021) [24]	24	COVID-19 automated diagnosis and risk assessment through metabolomics and machine learning
Cerqueira et al (2014) [25]	25	NICeSim: an open-source simulator based on machine learning techniques to support medical research on prenatal and perinatal care decision-making
das Neves et al (2020) [26]	26	Implementation of an expert system to determine eligibility and priorities for bone marrow transplants
Machado et al (2023) [27]	27	Multicenter Integrating radiomics, structured reports, and machine learning algorithms for assisted classification of COVID-19 in lung computed tomography
Razzouk et al (2006) [28]	28	Decision support system for the diagnosis of schizophrenia disorders
De Souza et al (2021) [29]	29	Leprosy screening based on artificial intelligence
Penha et al (2023) [30]	30	Single retinal image for diabetic retinopathy screening: performance of a handheld device with embedded artificial intelligence.
Goulart et al (2006) [31]	31	Artificial neural networks applied to study allergic conjunctivitis
de O Souza et al (2016) [32]	32	A screening system for smear-negative pulmonary tuberculosis using artificial neural networks.

### Risk of Bias

#### Overview

The assessment of risk of bias in the papers revealed the following classifications (Multimedia Appendix 2 [8-32]): 7 of 25 (28%) papers presented medium risk, 5 of 25 (20%) presented low risk, and 13 of 25 (52%) presented high risk [8-32]. Among the papers with high risk, 6 of 25 (46%) showed high risk in data analysis [24-26,29,31,32]. Paper 25 stood out with a high risk of bias in analyses, predictors, and outcomes [25]. To perform the PROBAST classification, the studies were categorized by risk domains: participants, predictors, outcome, and analysis. In each category, if the study presented an equivalent number of “N” or “?” and “Y,” it was classified as having a medium risk of bias. If “N” or “?” exceeded “Y,” the study was classified as high risk, whereas if “N” or “?” were fewer than “Y,” it was classified as low risk. Based on these four classifications, the prevailing category determined the overall risk classification of the study.

#### Participants

The lowest risk of bias among papers was in participant selection. For participant selection, 14 of 25 (56%) presented low risk (9, 11, 12, 14, 15, 17, 18, 20, 22, 26, 27, 28, 31, and 32), 8 of 25 (32%) presented medium risk (8, 13, 16, 19, 24, 25, 29, and 30), and 3 of 25 (12%) presented high risk (10, 21, and 23) [8-32].

#### Outcomes

For outcomes, 13 of 25 (52%) presented low risk (9, 10, 12, 13, 15, 16, 18, 19, 21, 22, 23, 29, and 30), 10 of 25 (40%) presented medium risk (8, 11, 14, 17, 20, 26, 27, 28, 31, and 32), and 3 of 25 (12%) presented high risk (8, 24, and 25) [8-32].

#### Analysis

The criterion with the highest risk of bias was data analysis, with 13 of 25 (52%) papers showing high risk (8, 14, 20, 21, 22, 23, 24, 25, 26, 27, 28, 29, and 30), 8 of 25 (32%) presenting medium risk (8, 9, 10, 11, 12, and 14), and 5 of 25 (20%) presenting low risk (15, 16, 17, 18, and 19) [8-32].

#### Predictors

For predictors, 7 of 25 (28%) presented low risk (9, 16, 18, 21, 23, 24, 27), 12 of 25 (48%) presented medium risk (8, 11, 12, 13, 15, 17, 19, 20, 22, 28, 30), and 8 of 25 (32%) presented high risk (10, 14, 25, 26, 29, 31, 32) [8-32].

### Study Objective

The studies’ objectives were classified as diagnosis or screening. Most (13/25, 52%) were focused on diagnosis, while 12 of 25 (48%) were focused on screening [8-32].

### Specialty

The medical areas covered by the tools are varied, with ophthalmology predominating, being the subject of 5 of 25 (20%) papers, with an emphasis on diabetic retinopathy diagnosis [8,13,14,30,31]. Next is infectology, covered in 4 of 25 (16%) papers, focusing on COVID-19 diagnosis [9,23,24,27]. Additionally, specialties such as cardiology, internal medicine, dermatology, pulmonology, and mastology are each the subject of 2 of 25 (8%) papers, totaling 10 of 25 (40%) of the papers [11,12,14,17-21,29,32]. Other specialties, such as allergy and immunology, endocrinology and metabolism, gastroenterology, neonatology, oncology, and psychiatry, are each covered in 1 of 25 (4%) papers, totaling 6 (24%) of the papers [10,15,22,25,26,28].

### Public or Private Initiative and Funding

The selected papers were characterized according to the nature of their initiative, being public or private. Most were part of a public initiative, totaling 17 of 25 (68%), while 7 of 25 (28%) were private initiatives, and 1 of 25 (4%) did not provide this information. Regarding funding, 7 of 25 (28%) papers did not provide information on this issue. In total, 2 of 25 (8%) papers were funded solely by the São Paulo Research Foundation (FAPESP), 2 of 25 (8%) by FAPESP in association with the National Council for Scientific and Technological Development (CNPQ), 1 of 25 (4%) by FAPESP in conjunction with various laboratories, 1 of 25 (4%) by FAPESP with the Technological Development Support Laboratory, 3 of 25 (12%) by the Minas Gerais State Research Support Foundation and CNPQ, and 1 of 25 (4%) by both and CAPES. Additionally, 1 of 25 (4%) was funded by CNPQ and the National Institutes of Health, 1 of 25 (4%) by MIT libraries, 1 of 25 (4%) by the Pontifical Catholic University of Paraná, 1 of 25 (4%) by Funding Authority for Studies and Projects and CAPES, and 2 of 25 (8%) by FAPERJ, with 1 in conjunction with National Institutes of Science and Technology and CNPQ and the other with CAPES. Finally, 1 of 25 (4%) was funded by Program to Support the Institutional Development of the Brazilian Unified Health System, and 1 of 25 (4%) had no financial support. This information demonstrates a predominance of funding, respectively, from CNPQ and FAPESP [8-32].

### Application Context or Location

Almost half of the papers were conducted in the state of São Paulo, totaling 12 of 25 (48%), of which 3 of 25 (25%) were conducted in collaboration with the states of Sergipe, Minas Gerais, and Amazonas [8,10,11,15,16,19-22,24,28,31]. Among these, 4 of 25 (33%) were associated with the Hospital das Clínicas of the University of São Paulo [10,21,24,28]. One of the studies also included the Hospital Estadual Sumaré, the Municipal Hospital of Paulínia, and the Delphina Rinaldi Abdel Aziz Hospital in Manaus [24]. The hospitals associated with Sergipe were unspecified, and the study in Minas Gerais was conducted at the Tropical Medicine Research Center in both states [11,16].

The other 4 of 25 (33%) studies conducted in the state of São Paulo took place at the São Camilo University Center, the Albert Einstein Israeli Hospital, the UNIFESP outpatient clinic, and an unspecified private health care institution [15,19,20,31].

In total, 2 of 25 (8%) tools from the papers were applied to populations during campaigns about diabetes mellitus, one in Santa Catarina and the other in Bahia [13,30]. A total of 3 of 25 (12%) studies were conducted in Rio de Janeiro, one at the Federal University of Rio de Janeiro (UFRJ) University Hospital, one at the Augusto Amaral Peixoto Polyclinic, and the other at Santa Casa de São Sebastião [12,25,32]. A total of 3 of 25 (12%) studies were conducted at the University Hospitals of UFRN (Rio Grande do Norte), UFPR (Paraná), and UFPE (Pernambuco) [9,14,26]. In total, 1 of 25 (4%) studies was conducted at the University Hospitals of Bahia, Paraíba, and Minas Gerais, associated with their respective federal universities [27]. Another 4 of 25 (16%) studies used data from the internet, with 1 of 4 (25%) associated with UFMG, 2 of 4 (50%) with UFRJ, and 1 of 4 (25%) with UFJF [17,18,23,29].

### AI Algorithms

The analyzed papers used different AI algorithms, classified into five distinct groups, with some studies combining more than one type in their development (Table 3). A total of 11 of 25 (44%) studies used machine learning, 9 of 25 (36%) used deep learning, 1 applied fuzzy Logic, 3 of 25 (12%) adopted ensemble methods, and 3 of 25 (12%) used expert systems [8-32].

**Table 3.** Type of AI^a^ and minimum requirements.

Study identification	Type of AI	Minimum requirements
8	ES^b^	Cell phone with sufficient memory and Android software version 4.0 (Ice Cream Sandwich) or higher.
9	DL^c^, ML^d^, EM^e^	Computer compatible with Windows, with sufficient memory and compatibility to run the machine learning models used.
10	ML	Computer compatible with Windows, with sufficient memory and compatibility to run the machine learning models used.
11	DL	ECG^f^ device connected to a Windows-compatible computer.
12	ML	Computer compatible with Windows, with sufficient memory and compatibility to run the machine learning models used.
13	DL	Specific cell phone model: Samsung Galaxy S10 (Android 11)
14	ML	Computer compatible with Windows, with sufficient memory and compatibility to run the machine learning models used.
15	ML, EM	Computer compatible with Windows, with sufficient memory and compatibility to run the machine learning models used.
16	DL	Specific cell phone model: Samsung Galaxy S10 (Android 11)
17	DL	Windows-compatible computer.
18	ML	Computer compatible with Windows, with sufficient memory and compatibility to run the machine learning models used.
19	DL	Android and iOS platforms for applicability, with no specific model requirement.
20	ML	Computer compatible with Windows, with sufficient memory and compatibility to run the machine learning models used.
21	FL^g^	Computer compatible with Windows, with sufficient memory and compatibility to run the machine learning models used.
22	ML	Computer compatible with Windows, with sufficient memory and compatibility to run the machine learning models used.
23	ML	Computer compatible with Windows, with sufficient memory and compatibility to run the machine learning models used.
24	ML	Computer compatible with Windows, with sufficient memory and compatibility to run the machine learning models used.
25	ML	Computer with specific software (NICeSim).
26	ES	Windows-compatible computer (Expert Sinta Software).
27	EM	Windows-compatible computer.
28	ES	Not specified
29	ML	Android and iOS platforms for applicability, with no specific model requirement.
30	DL	Computer compatible with Windows, with sufficient memory and compatibility to run the machine learning models used.
31	DL	Computer compatible with Windows, with sufficient memory and compatibility to run the machine learning models used.
32	DL	Computer compatible with Windows, with sufficient memory and compatibility to run the machine learning models used.

a AI: artificial intelligence.

b ES: Expert systems.

c DL: Deep learning.

d ML: Machine learning.

e EM: Ensemble methods.

f ECG: electrocardiogram.

g FL: Fuzzy logic.

### Minimum Requirements

Given the context of limited-income countries, such as Brazil, and their financial limitations, the minimum technological requirements necessary for the functionality of the tools were observed. The primary requirement is a computer, required by 19 of 25 (76%) of the papers, with 16 of 19 (84%) needing memory and compatibility for the machine learning models in these studies, 2 of 19 (10%) requiring specific software, and 1 of 19 (5%) with an electrocardiogram device connected to the computer, all compatible with Windows [8,10-12,14,15,17,18,20-27,30-32].

Additionally, 3 of 25 (12%) required specific cell phone models: a phone with sufficient memory and Android software version 4.0 (Ice Cream Sandwich; paper 8) and Samsung Galaxy S10 (Android 11; papers 13 and 14) [9,13,14]. A total of 2 (8%) were applicable on Android and iOS platforms, with no specific device model requirements (papers 19 and 29) [19,29]. Another 1 of 25 (4%) papers did not specify the necessary technology [28].

### Test or External Application or Evidence of Health Improvement

All the papers conducted some form of testing to evaluate and validate the tools. However, only two of them performed a second evaluation with application to a different population. Additionally, all studies presented some evidence of health improvement, but the risks of bias associated with these evidences will be explored [8-32].

### Results of Statistical Analyses

**Figure 2.**
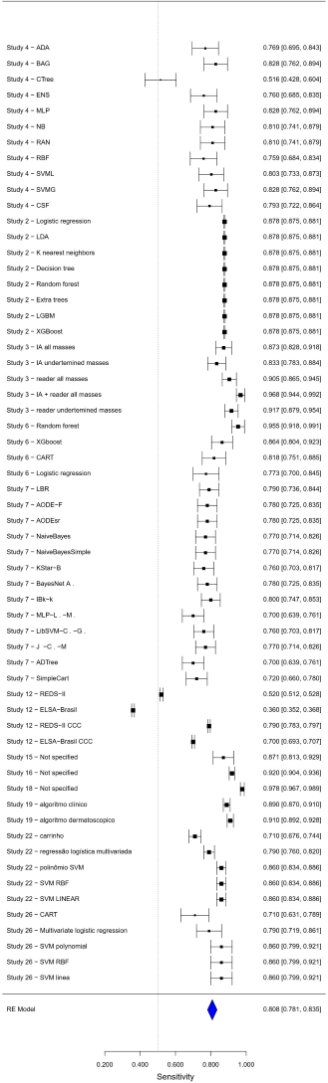
Forest plot sensitivity.

The sensitivity meta-analysis, using a random-effects model, resulted in a combined estimate of 0.8113 (95% CI 0.7856‐0.8369), with a *z* score value of 62.0035 (*P*<.001). The analysis revealed high heterogeneity among the studies (*I*²=88%), suggesting significant variability in the results of the included studies. The forest plot in Figure 2 visualizes each study’s sensitivity estimate and the combined estimate, highlighting the dispersion of the results.

**Figure 3.**
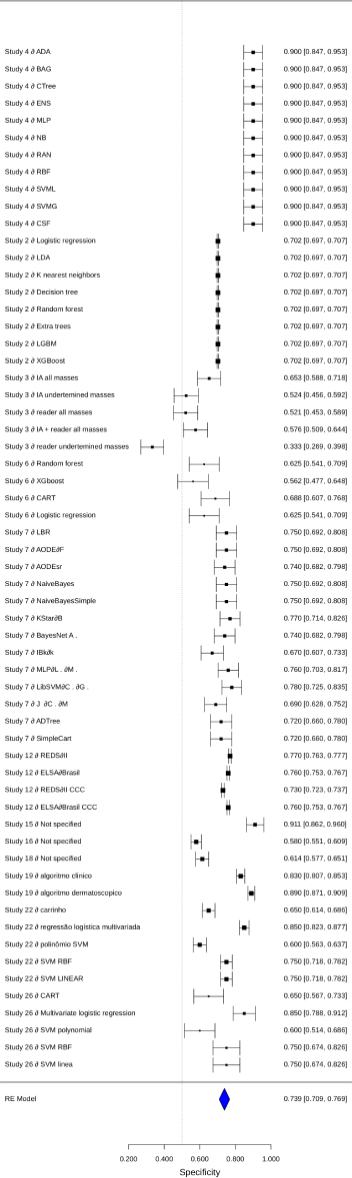
Forest plot specificity.

The combined specificity estimate was 0.7417 (95% CI 0.7113‐0.7722), with a *z* score value of 47.7605 (*P*<.001). The analysis indicated high heterogeneity (*I*²=88%), as shown in Figure 3, reflecting substantial differences in the study results. The corresponding forest plot illustrates these individual estimates and the combined estimate from the meta-analysis.

**Figure 4.**
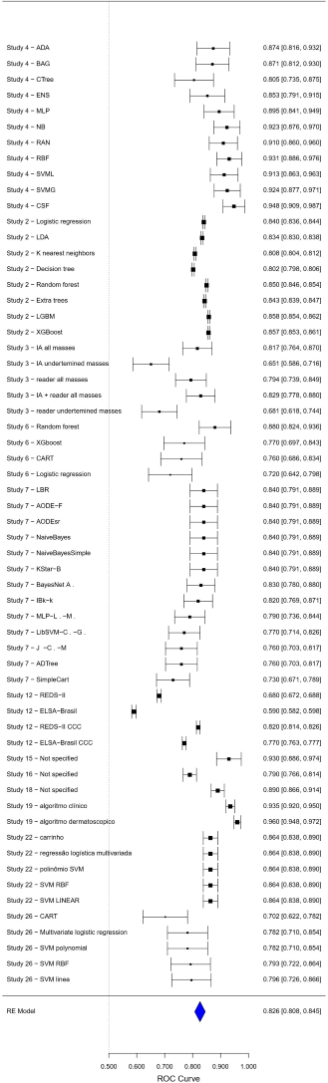
Forest plot for area under the receiver operating characteristic (ROC) curve.

The AUC meta-analysis presented a combined estimate of 0.8308 (95% CI 0.8124‐0.8492), with a *z* score value of 88.4640 (*P*<.001). The heterogeneity was moderate to high (*I*²=76%), as visualized in the forest plot in Figure 4, suggesting variations in methods or populations among the studies.

The forest plots (Figures3  4) provide a visual representation of the individual and combined estimates for sensitivity, specificity, and AUC. Each graph highlights the variability among the studies and the combined estimate obtained through the meta-analysis. The dashed vertical line in the graphs represents the combined estimate, while the CIs are indicated by horizontal bars for each study. Multimedia Appendix 3 presents the compilation of sensitivities, specificities, and the AUC.

## Discussion

### Principal Results

This study demonstrates that AI tools in Brazilian health are widely used for diagnosis and screening, with emphasis on ophthalmology and infectious diseases, and the public initiative is preponderant in the development of these technologies. In this context, the application of AI in health care has advanced significantly in Brazil, with various tools being developed for screening and diagnosis purposes. This distinction between screening and diagnosis is essential, as it directly influences the implementation and impact of technologies in different health care contexts. The primary goal of screening tools is to identify individuals at risk of a given condition, prioritizing sensitivity maximization. This means these tools are designed to ensure that few actual cases go unnoticed, even if it increases false positives. This approach is fundamental in scenarios where early detection can save lives or prevent disease progression [33].

On the other hand, diagnosis tools aim to confirm or rule out the presence of a condition in individuals already identified as at risk. In these cases, accuracy becomes crucial, with a more careful balance between sensitivity and specificity. Often, there is a focus on maximizing specificity to reduce false positives, thus avoiding unnecessary treatments. These accuracy requirements reflect the distinct needs of screening and diagnosis tools, with screening being more permissive regarding false positives to ensure no actual case is overlooked, while diagnosis seeks high accuracy to prevent clinical errors [33].

The implementation of these tools also varies depending on the context of use. Screening tools are often used in resource-limited settings, where there is a need to process large volumes of data quickly. Thus, they are used in community screening campaigns or public health programs, where the focus is on quickly identifying cases that need more thorough evaluation, as shown in papers 12, 13, 16, 22, 30, and 32 [12,13,16,22,23,32]. Diabetic retinopathy screening is prominent in this context, covered in papers 13, 16, and 30, as well as tuberculosis in papers 12 and 32, conditions highlighted in the Brazilian context [12,13,16,30,32]. Rare syndromes that require early intervention, such as inborn immunity errors in paper 22, were also explored [22].

Highlighted specialties for AI tools include ophthalmology and infectious disease. The former benefits from AI’s image analysis capabilities, revisiting the topic discussed earlier: diabetic retinopathy research (13, 16, 30), allergic conjunctivitis screening (31), and glaucoma diagnosis (9) [8,13,16,30,31]. Meanwhile, infectious diseases, critically important in Brazil, stood out in COVID diagnosis—a crucial aspect, given the global impact of the pandemic. In this case, tools were used for clinical diagnosis (23,24), with one using imaging diagnostics through computed tomography (27) [23,24,27]. Another area worth noting, not due to the number of tools produced but because of its clinical importance in Brazil, also within infectious disease, is pulmonology, which includes tuberculosis screening, with both papers covering testing and clinical symptoms (12 and 32) [12,32].

The predominance of public initiatives and funding from scientific development programs—particularly National Council for Scientific and Technological Development (CNPQ) and São Paulo Research Foundation (FAPESP)—highlights the importance of state support for research. This support is crucial for advancing tools and studies in health care, significantly benefiting the country. Furthermore, the presence of private initiatives reflects the ongoing need for health care technology advancement in Brazil. Public-private partnerships, exemplified by Program to Support the Institutional Development of the Brazilian Unified Health System, are essential for improving practices and innovations in the sector, fostering a constantly evolving environment [8-32].

Brazil’s vast and diverse territory shows varying trends in areas such as infrastructure, access to technology, and development in health care and educational institutions [34]. This review revealed a significant concentration of studies conducted in São Paulo state (12/25, 48%), with the Hospital das Clínicas at the University of São Paulo being involved in 33% (4/25) of these studies [8,10,11,15,16,19-22,24,28,31]. This event highlights the tendency for AI use to be concentrated in the country’s wealthiest state: São Paulo. Additionally, a notable collaboration between São Paulo and other states, such as Sergipe, Minas Gerais, and Amazonas, highlights joint efforts to develop technology across regional boundaries within the country [11,16,24].

This is essential as it allows adaptation to different realities, with broader applications and more effective tools (tested in various populations). 

Other states, such as Rio Grande do Norte, Bahia, Santa Catarina, and Pernambuco, also produced AI tools, demonstrating broad participation across Brazilian states [9,13,14,30]. Finally, prominent institutions, including Federal University of Minas Gerais (UFMG), UFRJ, and Federal University of Juiz de Fora (UFJF), used internet data to develop their technologies, showcasing the importance of digital resources for health research, expanding the reach and diversity of studied populations [17,18,23,29].

The most commonly used AI algorithm was machine learning (11/25, 44%), which uses models attempting to predict future outcomes based on a dataset and is helpful in automated diagnostics and decision-support systems, as seen in papers 7 and 25 [10,14,35]. Deep learning, a subcategory of machine learning, appeared in 36% (9/25) of the studies, allowing more complex data analysis involving larger volumes and image recognition, a critical feature in papers 18 and 22, for example, for image evaluation [13,23,36]. Additionally, paper 15 used ensemble methods, which combine multiple machine learning models to improve decision accuracy [27,37].

Another important algorithm is expert systems, which operate based on a predefined set of rules and knowledge, used for decision support, as in paper 26, or diagnosis, as in paper 8 [9,26]. Finally, fuzzy logic is valuable for decision-making in less precise cases requiring flexible interpretation, such as breast cancer characterization in paper 21 [21].

In limited-income countries like Brazil, minimum technological requirements for implementing AI tools in health care should be carefully assessed due to financial constraints and frequently inadequate technological infrastructure. The analysis of minimum requirements, as presented, reveals a predominant reliance on computers as a fundamental platform, with 76% (19/25) of papers indicating their necessity, underscoring the importance of computing in executing machine learning models [8,10-12,14,15,17,18,20-27,30-32].

The need for computers compatible with Windows operating systems was observed in 18 of 25 (72%) papers, revealing this operating system’s prevalence in health care environments in Brazil. The use of specific software for running AI models was also noted, though at a lower rate (2/19, 10%). The compatibility with Windows may be attributed to its wide adoption and familiarity in the Brazilian market, potentially reducing barriers to adopting new technologies [8,10,11,14,17,20,22-27,30-32].

Besides computers, one paper mentioned using electrocardiogram devices linked to computers, indicating additional hardware integration for data monitoring and collection [14]. The need for specific mobile devices was identified in 3 of 25 (12%) papers, focusing on phones with minimum specifications, such as sufficient memory and specific Android OS versions. This requirement for compatibility with particular versions may limit the applicability of tools on older or less common mobile devices, potentially restricting technology access and dissemination, a negative point.

The diverse application of tools across Android and iOS platforms, without strict device model specifications, reflects an attempt at greater flexibility and accessibility [19,29].

Regarding ethics, AI models must be prepared to withstand cyberattacks to ensure the security and protection of patient data, with tools similar to those used by the pharmaceutical industry being a possible alternative. Furthermore, although much of the data are already stored for program reporting, policy development, strategic planning, and advocacy purposes, it often remains inaccessible to research areas. In this context, improving access to these data requires, first and foremost, maintaining clear communication with the population to ensure a proper understanding of how their data will be used, the potential benefits, and the measures in place to protect their privacy. Transparent dialog, community engagement, and the development of context-sensitive consent processes are essential to building public trust and fostering an ethical data-sharing environment [1].

The meta-analysis on AI model performance in medical diagnostics and screening revealed important clinical insights. The combined sensitivity was 0.8113, indicating that, on average, AI models correctly identify approximately 81% of true positives. This high sensitivity is crucial in clinical settings, particularly in initial screening, as it helps ensure that most patients with a medical condition are identified and treated, minimizing the risk of false negatives.

The combined specificity was 0.7417, suggesting that AI models correctly identify around 74% of true negatives. Although this specificity is considered good, it is lower than sensitivity, which may indicate a tendency for false positives in some scenarios. This is relevant, as false positives can lead to unnecessary treatments or additional tests for patients who do not have the condition in question. Due to existing financial constraints, these potential unnecessary costs are harmful, especially in low-resource settings.

The combined area under the AUC was 0.8308, pointing to AI models’ excellent discriminatory ability between positive and negative cases. The AUC reflects overall model performance, demonstrating a satisfactory balance between sensitivity and specificity, which is essential for accurate diagnostics and screening.

### Limitations

All papers presented evidence of health improvement, suggesting that AI tools have the potential to positively impact clinical outcomes. However, testing is crucial to ensure the effectiveness and safety of AI tools in health care. All papers reviewed reported some form of testing to validate their tools, indicating a commitment to performance evaluation of the proposed solutions.

The papers’ main limitation was data analysis, with criteria Q15, Q16, and Q17 being absent in almost all studies. This lack of information regarding how the analysis was conducted suggests a significant bias, with potential participants being excluded without justification and unmentioned complexities, which could lead to biased estimates. Therefore, for future research, methodological transparency must be prioritized.

Among the outcomes, most presented a medium risk of bias, mainly due to missing data in criteria Q10 and Q11, which assess whether the evaluator was aware of the predictors when determining the outcome, potentially inflating the reported accuracy measures, or whether there was a time interval between predictor assessment and outcome determination.

In the predictors, a crucial issue not reported in most papers was Q4: Were predictor assessments made without knowledge of outcome data? Since this information was missing, it is possible that predictor collection was biased, influencing the actual effectiveness of the tools, compromising reproducibility, and the critical evaluation of the results presented.

Few studies had an inadequate participant selection process and sample representativeness, limiting generalizability to distinct populations, especially in a country as diverse as Brazil.

For future studies, the key is greater transparency with the data to increase trust in the tools.

Nevertheless, a major limitation of the studies is that only 3 papers conducted a second evaluation by applying the tool to a different population, limiting the generalizability of the results and increasing the possibility of statistical errors in real-world population outcomes. This practice raises the risk of overfitting, where the model performs excellently on the original sample but fails when applied to new contexts. One possibility for future studies is to conduct the test in different centers, with partnerships between universities, to increase the sample size and the tool’s potential.

Another relevant factor is the high heterogeneity observed in all analyses, with *I*² values ranging from 76% to 88%. This variability suggests that the effectiveness of AI models may vary significantly between different studies and contexts. Differences in algorithms used, study populations, and implementation methods may contribute to this variation, further reinforcing the concern about retesting these tools in other contexts.

### Conclusions

This review demonstrates a broad application of AI technologies in diagnostic and treatment areas, with relative equality in the use of both types of tools and broad use across Brazil in varied contexts. The predominance of public funding indicates the potential for tools for use in the Brazilian UHS, which is significant due to the widespread reliance on the Brazilian public health care system. The variety of specialties highlights the diversity of AI applications and their importance in the health sector. Finally, the need for secondary testing points to future research opportunities.

## Supplementary material

10.2196/69547 Multimedia Appendix 1 Search strings.

10.2196/69547 Multimedia Appendix 2 Classifications of assessment of risk of bias (Y=Yes, N=No, and ?=Without information).

10.2196/69547 Multimedia Appendix 3 Sensitivity, specificity, and area under the curve (AUC).

10.2196/69547 Checklist 1 PRISMA 2020 checklist.

## Abbreviations

AI: artificial intelligence
AUC: area under the receiver operating characteristic curve
CNPQ: National Council for Scientific and Technological Development
FAPESP: São Paulo Research Foundation
PRISMA: Preferred Reporting Items for Systematic Reviews and Meta-Analyses
PROBAST: Prediction model Risk Of Bias Assessment Tool
UFRJ: Federal University of Rio de Janeiro
UHS: Unified Health System
