# Supplementary material for: Diagnostic and Screening AI Tools in Brazil’s Resource-Limited Settings: Systematic Review
Source: JMIR AI. 2025 Sep 10;4:e69547. doi: 10.2196/69547 (PMC12422524; doi:10.2196/69547)
Supplement: Multimedia Appendix 1 [file ai-v4-e69547-s001.pdf]

	PUBMED	20/11/2023 20:17
#1	(((((((primary health care[MeSH Terms]) OR (public health practice[MeSH Terms])) OR (community health centers[MeSH Terms])) OR (Rural Health Services[MeSH Terms])) OR (Preventive Health Services[MeSH Terms])) OR (Schools, Public Health[MeSH Terms])) OR (Urban Health Services[MeSH Terms])) OR (Suburban Health Services[MeSH Terms])	1.366.018
#2	((((((Healthcare Disparities[MeSH Terms]) OR (Minority Health[MeSH Terms]))) OR (Health Status Disparities[MeSH Terms])) OR (Access to Primary Care[MeSH Terms])) OR (Socioeconomic Disparities in Health[MeSH Terms])) OR (Access Essential Medicines Health Technologies) OR (Healthcare Models)	308.408
#3	(((Physicians, Primary Care[MeSH Terms]) OR (Physicians, Family[MeSH Terms])) OR (Students, Health Occupations[MeSH Terms])) OR (Physicians[MeSH Terms])	265.432
#4	#1 OR #2 OR #3	1.847.797
#5	(Artificial Intelligence[MeSH Terms]) OR (Artificial Intelligence)	217.724
#6	(brazil[MeSH Terms]) OR (brazil)	506.549
#12	#4 AND #5 AND #6	233

	Scielo	20/11/2023 20:37
#1	"primary health care" OR "public health practice" OR "community health centers" OR "Rural Health Services" OR "Preventive Health Services" OR "Schools, Public Health" OR "Urban Health Services" OR "Suburban Health Services"	12.012
#2	("Healthcare Disparities") OR ("Minority Health") OR ("Health Status Disparities") OR ("Access to Primary Care") OR ("Socioeconomic Disparities in Health") OR	553

	("Access to Essential Medicines Health and Technologies") OR ("Healthcare Models")	
#3	("Physicians, Primary Care") OR ("Physicians, Family") OR ("Students, Health Occupations") OR ("Physicians")	6.015
#4	#1 OR #2 OR #3	17.785
#5	("Artificial Intelligence") OR ("Inteligência artificial") OR ("inteligência artificial") OR ("artificial intelligence")	1.044
#6	(Brazil) OR (Brasil) OR (brazil) OR (brasil)	81.577
#7	#4 AND #5 AND #6	6

	Lilacs	20/11/2023 20:45
#1	(atenção primária à saúde) OR (prática de saúde pública) OR (assistência à saúde) OR (faculdades de saúde pública) AND (db:("LILACS"))	84.200
#2	(disparidades em assistência à saúde) OR (saúde das minorias) OR (disparidades nos níveis de saúde) OR (acesso aos serviços de saúde) OR (disparidades socioeconômicas em saúde) OR (acesso a medicamentos essenciais e tecnologias em saúde) OR (modelos de assistência à saúde) AND (db:("LILACS"))	11.020
#3	(médicos de atenção primária) OR (médicos de família) OR (estudantes de ciências da saúde) OR (médicos) AND (db:("LILACS"))	47.316
#4	#1 OR #2 OR #3	122.287
#5	(artificial intelligence) OR (inteligência artificial) OR (inteligência artificial) OR (artificial intelligence) AND (db:("LILACS"))	561
#6	(Brazil) OR (Brasil) OR (brazil) OR (brasil)	136.392
#7	#4 AND #5 AND #6	14

	Cochrane	20/11/2023 20:54
#1	MeSH descriptor: [Primary Health Care] explode all trees	10445
#2	MeSH descriptor: [Public Health Practice] explode all trees	19898
#3	MeSH descriptor: [Rural Health Services] explode all trees	416
#4	MeSH descriptor: [Preventive Health Services] explode all trees	41815
#5	MeSH descriptor: [Schools, Public Health] explode all trees	3
#6	MeSH descriptor: [Urban Health Services] explode all trees	161
#7	MeSH descriptor: [Suburban Health Services] explode all trees	6
#8	#1 OR #2 OR #3 OR #4 OR #5 OR #6 OR #7	54827
#9	MeSH descriptor: [Healthcare Disparities] explode all trees	277
#10	MeSH descriptor: [Minority Health] explode all trees	28
#11	MeSH descriptor: [Health Status Disparities] explode all trees	254
#12	MeSH descriptor: [Health Services Accessibility] explode all trees	1499
#13	#9 OR #10 OR #11 OR #12	1969
#14	MeSH descriptor: [Physicians, Primary Care] explode all trees	214
#15	MeSH descriptor: [Physicians, Family] explode all trees	511
#16	MeSH descriptor: [Students, Health Occupations] explode all trees	2621
#17	MeSH descriptor: [Physicians] explode all	3287

	trees	
#18	#14 OR #15 OR #16 OR #17	5844
#19	#8 OR #13 OR #18	60846
#20	MeSH descriptor: [Artificial Intelligence] explode all trees	2958
#21	Artificial Intelligence	1948
#22	#20 OR #21	4297
#23	#19 AND #22	179
#24	(Brazil)	18788
#25	brasil OR brazil	19211
#26	MeSH descriptor: [Brazil] explode all trees	2345
#27	#24 OR #25 OR #26	19211
#28	#23 AND #27	1

	Embase	20/11/2023 21:10
#1	(((((((primary AND ('health'/exp OR health) AND ('care'/exp OR care) OR 'public'/exp OR public) AND ('health'/exp OR health) AND ('practice'/exp OR practice) OR 'community'/exp OR community) AND ('health'/exp OR health) AND centers OR 'rural'/exp OR rural) AND ('health'/exp OR health) AND services OR preventive) AND ('health'/exp OR health) AND services OR 'schools,'/exp OR schools,) AND ('public'/exp OR public) AND ('health'/exp OR health) OR urban) AND ('health'/exp OR health) AND services OR suburban) AND ('health'/exp OR health) AND services	84.634
#2	((((('health care disparity' OR minority) AND health OR health) AND status AND disparities OR access) AND to AND primary AND care OR socioeconomic) AND disparities AND in AND health OR access) AND to AND essential AND medicines AND health AND technologies OR healthcare) AND models	78.461

#3	((physicians, AND primary AND care OR physicians,) AND family OR students,) AND health AND occupations OR physicians	649.481
#4	#1 OR #2 OR #3	801.538
#5	((artificial AND intelligence OR inteligência) AND artificial OR inteligência) AND artificial OR artificial) AND intelligence	97.368
#6	brazil OR brasil	816.903
#7	#4 AND #5 AND #6	94
#8	#4 AND #5 AND #6 AND [embase]/lim	72

	Web of Science	20/11/2023 21:56
#1	(((((((ALL=(primary health care)) OR ALL=(public health practice)) OR ALL=(community health centers)) OR ALL=(Rural Health Services)) OR ALL=(Preventive Health Services)) OR ALL=(Schools, Public Health)) OR ALL=(Urban Health Services)) OR ALL=(Suburban Health Services)	688.966
#2	((((((ALL=(Healthcare Disparities)) OR ALL=(Minority Health)) OR ALL=(Health Status Disparities)) OR ALL=(Access to Primary Care)) OR ALL=(Socioeconomic Disparities in Health)) OR ALL=(Access to Essential Medicines Health and Technologies)) OR ALL=(Healthcare Models)	323.925
#3	(((ALL=(Physicians, Primary Care)) OR ALL=(Physicians, Family)) OR ALL=(Students, Health Occupations)) OR ALL=(Physicians)	5.350.882
#4	#1 OR #2 OR #3	6.167.194
#5	ALL=(Artificial Intelligence)	460.477
#6	(((ALL=(Brazil)) OR ALL=(brasil)) OR ALL=(Brasil)) OR ALL=(brazil)	1.438.291
#7	#4 AND #5 AND #6	368
