# Supplementary material for: Diagnostic and Screening AI Tools in Brazil’s Resource-Limited Settings: Systematic Review
Source: JMIR AI. 2025 Sep 10;4:e69547. doi: 10.2196/69547 (PMC12422524; doi:10.2196/69547)
Supplement: Multimedia Appendix 2 [file ai-v4-e69547-s002.docx]

| AUTHOR | NAME | QV1 | QV2 | QV3 | QV4 | QV5 | QV6 | QV7 | QV8 | QV9 | QV10 | QV11 | QV12 | QV13 | QV14 | QV15 | QV16 | QV17 | QV18 | QV19 | RISK |
| --- | --- | --- | --- | --- | --- | --- | --- | --- | --- | --- | --- | --- | --- | --- | --- | --- | --- | --- | --- | --- | --- |
| Araujo et al. (2022) [9] | DZC DIAG: mobile application based on expert system to aid in the diagnosis of dengue, Zika, and chikungunya | Y | ? | Y | N | Y | Y | Y | N | Y | N | N | Y | Y | Y | ? | ? | ? | Y | ? | MEDIUM |
| Shigueokal et al. (2018) [8] | Automated algorithms combining structure and function outperform general ophthalmologists in diagnosing gl oma | Y | Y | Y | Y | Y | Y | Y | N | Y | Y | Y | Y | Y | Y | ? | ? | ? | Y | ? | MEDIUM |
| Tenório et al. (2011) [10] | Artificial intelligence techniques applied to the development of a decision-support system for diagnosing celiac disease | N | N | ? | ? | ? | Y | Y | Y | Y | Y | ? | Y | Y | ? | ? | ? | ? | Y | Y | MEDIUM |
| Oliveira et al. (2021) [11] | Left ventricular systolic dysfunction predicted by artificial intelligence using the electrocardiogram in Chagas disease patients–The SaMi-Trop cohort. | Y | Y | Y | ? | Y | Y | ? | Y | Y | ? | ? | Y | Y | Y | ? | N | ? | Y | ? | MEDIUM |
| Souza et al. (2018) [12] | Screening for active pulmonary tuberculosis: Development and applicability of artificial neural network models | Y | Y | Y | N | Y | Y | Y | Y | Y | ? | ? | Y | ? | Y | ? | ? | ? | Y | Y | MEDIUM |
| Malerbi et al. (2021) [13] | Diabetic Retinopathy Screening Using Artificial Intelligence and Handheld Smartphone-BasedRetinal Camera | N | Y | Y | ? | Y | Y | Y | Y | Y | ? | ? | Y | ? | Y | ? | ? | Y | Y | ? | MEDIUM |
| Albuquerque et al. (2023) [14] | Osteoporosis screening using machine learning and electromagnetic waves | Y | Y | Y | ? | ? | Y | Y | N | Y | ? | ? | Y | ? | Y | ? | Y | ? | Y | ? | MEDIUM |
| Tavares et al.(2022) [15] | Prediction of metabolic syndrome: A machine learning approach to help primary prevention | Y | Y | Y | ? | Y | Y | Y | N | Y | Y | Y | Y | Y | Y | Y | ? | ? | Y | ? | LOW |
| Angélica et al. (2023) [16] | Clinical validation of a smartphone-based retinal camera for diabetic retinopathy screening | N | Y | Y | Y | Y | Y | Y | ? | Y | ? | Y | Y | Y | Y | ? | ? | Y | Y | Y | LOW |
| Jidling et al. (2023) [17] | Screening for Chagas disease from the electrocardiogram using a deep neural network | Y | Y | Y | N | Y | Y | Y | N | Y | N | ? | Y | Y | N | Y | Y | Y | Y | Y | LOW |
| Filho et al. (2020) [18] | Can machine learning be useful as a screening tool for depression in primary care? | Y | Y | Y | Y | Y | Y | Y | Y | Y | Y | ? | Y | Y | Y | Y | ? | Y | Y | Y | LOW |
| Giavina-Bianchi et al. (2021) [19] | Implementation of artificial intelligence algorithms for melanoma Screening in a primary care setting | Y | ? | Y | ? | Y | Y | Y | ? | Y | ? | Y | Y | Y | Y | ? | ? | ? | Y | Y | LOW |
| Fleury et al. (2020) [20] | Impact of radiomics on the breast ultrasound radiologist’s clinical practice: from lumpologist to data wrangler | Y | Y | Y | Y | ? | Y | Y | ? | Y | ? | ? | Y | Y | N | Y | ? | ? | ? | ? | HIGH |
| Miranda et al. (2015) [21] | Computer-aided diagnosis system based on fuzzy logic for breast cancer categorization | N | ? | Y | Y | Y | Y | Y | Y | Y | Y | ? | Y | Y | ? | ? | ? | ? | N | ? | HIGH |
| Takao et al. (2022) [22] | Artificial Intelligence in Allergy and Immunology: Comparing Risk Prediction Models to Help Screen Inborn Errors of Immunity. | Y | Y | Y | ? | Y | Y | Y | Y | Y | ? | ? | Y | Y | N | ? | ? | ? | Y | ? | HIGH |
| Marques et al. (2023) [23] | An online platform for COVID-19 diagnostic screening using a machine learning algorithm | N | N | Y | Y | Y | Y | Y | ? | Y | ? | Y | Y | ? | Y | N | ? | N | ? | Y | HIGH |
| Delafiori et al. (2021) [24] | Covid-19 Automated Diagnosis and Risk Assessment through Metabolomics and Machine Learning | N | Y | Y | Y | Y | Y | Y | ? | ? | ? | ? | Y | ? | ? | ? | ? | ? | ? | Y | HIGH |
| Cerqueira et al. (2014) [25] | NICe: an open-source simulator based on machine learning techniques to support medical research on prenatal and perinatal care decision making | Y | ? | ? | ? | ? | Y | ? | ? | ? | ? | ? | Y | ? | ? | ? | ? | ? | Y | ? | HIGH |
| Neves et al. (2001) [26] | Implementation of an expert system to determine eligibility and priorities for bone marrow transplants | Y | Y | ? | ? | ? | Y | Y | ? | Y | ? | ? | Y | ? | ? | ? | ? | ? | Y | ? | HIGH |
| Machado et al. (2023) [27] | Multi-center Integrating Radiomics, Structured Reports, and Machine Learning Algorithms for Assisted Classification of COVID-19 in Lung Computed Tomography | Y | Y | Y | Y | Y | Y | Y | ? | Y | ? | ? | Y | ? | ? | ? | ? | ? | Y | ? | HIGH |
| Razzouk et al. (2021) [28] | Decision support system for the diagnosis of schizophrenia disorders | Y | Y | Y | ? | Y | Y | Y | ? | Y | ? | ? | ? | ? | ? | ? | ? | ? | Y | ? | HIGH |
| De Souza et al. (2021) [29] | Leprosy Screening Based on Artificial Intelligence | Y | ? | ? | ? | ? | Y | Y | N | Y | Y | ? | Y | Y | ? | Y | ? | ? | ? | ? | HIGH |
| Penha et al. (2023) [30] | Single retinal image for diabetic retinopathy | Y | ? | Y | ? | Y | Y | Y | Y | Y | Y | Y | Y | ? | N | ? | ? | ? | Y | ? | HIGH |
| Goulart et al. (2006) [31] | Artificial neural networks applied to study allergic conjunctivitis | Y | Y | Y | ? | Y | Y | ? | N | Y | ? | ? | Y | Y | ? | ? | ? | ? | ? | ? | HIGH |
| De Souza et al. (2016) [32] | A screening system for smear-negative pulmonary tuberculosis | Y | Y | Y | ? | N | Y | ? | N | Y | ? | Y | Y | ? | Y | ? | N | ? | Y | ? | HIGH |
