# Supplementary material for: Diagnostic and Screening AI Tools in Brazil’s Resource-Limited Settings: Systematic Review
Source: JMIR AI. 2025 Sep 10;4:e69547. doi: 10.2196/69547 (PMC12422524; doi:10.2196/69547)
Supplement: Multimedia Appendix 3 [file ai-v4-e69547-s003.docx]

| ID | AUC | Sensitivity | Specificity |
| --- | --- | --- | --- |
| 8 |  | 0,9688 | 0 |
| 9 (ADA) | 0,874 | 0,77 | 0,90 |
| 9 (BAG) | 0,871 | 0,83 | 0,90 |
| 9 (CSF1) | 0,948 | 0,79 | 0,90 |
| 9 (CTree) | 0,805 | 0,52 | 0,90 |
| 9 (ENS) | 0,853 | 0,76 | 0,90 |
| 9 (MLP) | 0,895 | 0,83 | 0,90 |
| 9 (NB) | 0,923 | 0,81 | 0,90 |
| 9 (RAN) | 0,91 | 0,81 | 0,90 |
| 9 (RBF) | 0,931 | 0,76 | 0,90 |
| 9 (SVMG) | 0,924 | 0,83 | 0,90 |
| 9 (SVML) | 0,913 | 0,80 | 0,90 |
| 10 (LBR) | 0,84 | 0,79 | 0,75 |
| 10 (ADTree) | 0,76 | 0,70 | 0,72 |
| 10 (AODE-F1) | 0,84 | 0,78 | 0,75 |
| 10 (AODEsr) | 0,84 | 0,78 | 0,74 |
| 10 (BayesNet A0.75) | 0,83 | 0,78 | 0,74 |
| 10 (IBk-k16) | 0,82 | 0,80 | 0,67 |
| 10 (J48-C0.7-M9) | 0,76 | 0,77 | 0,69 |
| 10 (KStar-B60) | 0,84 | 0,76 | 0,77 |
| 10 (LibSVM-C1.0-G0.0) | 0,77 | 0,76 | 0,78 |
| 10 (MLP-L0.4-M0.2) | 0,79 | 0,70 | 0,76 |
| 10 (NaiveBayes) | 0,84 | 0,77 | 0,75 |
| 10 (NaiveBayesSimple) | 0,84 | 0,77 | 0,75 |
| 10 (SimpleCart) | 0,73 | 0,72 | 0,72 |
| 11 |  | 0,73 | 0,83 |
| 12 | 0,79 | 0,92 | 0,58 |
| 13 | 0.89 | 0,98 | 0,61 |
| 14 |  |  |  |
| 14 Decision tree | 0,8233 |  |  |
| 14 Extra tree | 0,869 |  |  |
| 14 Extra tree | 0,7444 |  |  |
| 14 Gaussian naive bayes | 0,7497 |  |  |
| 14 Hist grad boosting | 0.8565 |  |  |
| 14 K neighbors | 0,8002 |  |  |
| 14 Linear SVC | 0,743 |  |  |
| 14 Stochastic gradient desc | 0,7541 |  |  |
| 14 XG boost | 0,8754 |  |  |
| 14 XG boost RF | 0,8310 |  |  |
| 14 Ada boost | 0,8404 |  |  |
| 14 Bagging | 0,8505 |  |  |
| 14 Gaussian process | 0,8509 |  |  |
| 14 Gradient boosting | 0,8688 |  |  |
| 14 Linear discriminant | 0,8052 |  |  |
| 14 Logistic regression | 0,8037 |  |  |
| 14 Quadratic discrimant | 0,7858 |  |  |
| 14 Random forest | 0,8943 |  |  |
| 14 SVC | 0,7531 |  |  |
| 15 (Decision tree) | 0,802 | 0,88 | 0,70 |
| 15 (Extra trees) | 0,843 | 0,88 | 0,70 |
| 15 (K nearest neighbors) | 0,808 | 0,88 | 0,70 |
| 15 (LDA) | 0,834 | 0,88 | 0,70 |
| 15 (LGBM) | 0,858 | 0,88 | 0,70 |
| 15 (Logistic regression) | 0,84 | 0,88 | 0,70 |
| 15 (Random forest) | 0,85 | 0,88 | 0,70 |
| 15 (XGBoost) | 0,857 | 0,88 | 0,70 |
| 16 (Referable DR) intermodality |  | 0,91 | 0,81 |
| 16 (Vision threatening DR) intermodality |  | 0,87 | 0,83 |
| 17 (ELSA-Brasil CCC) | 0,77 | 0,70 | 0,76 |
| 17 (ELSA-Brasil) | 0,59 | 0,36 | 0,76 |
| 17 (REDS-II CCC) | 0,82 | 0,79 | 0,73 |
| 17 (REDS-II) | 0,68 | 0,52 | 0,77 |
| 18 (model AB) | 0,58 | 0,90 |  |
| 18 (model CART) | 0,65 | 0,90 |  |
| 18 (model GB) | 0,74 | 0,90 |  |
| 18 (model KNN) | 0,81 | 0,90 |  |
| 18 (model LR) | 0,61 | 0,90 |  |
| 18 (model RF) | 0,87 | 0,90 |  |
| 18 (model SVM) | 0,61 | 0,90 |  |
| 18 (model XGB) | 0,77 | 0,90 |  |
| 19 (clinical algorithm) | 0,935 | 0,89 | 0,83 |
| 19 (dermoscopic algorithm) | 0,96 | 0,91 | 0,89 |
| 20 (all masses) | 0,817 | 0,87 | 0,65 |
| 20 (IA + reader) (all masses) | 0,829 | 0,97 | 0,58 |
| 20 (IA + reader)(undertemined masses) | 0,702 | 1,00 | 0,29 |
| 20 (undertemined masses) | 0,651 | 0,83 | 0,52 |
| 21 (calcifications) | NA | 0,05 | 0,02 |
| 21(nodules) | - | 0,76 | 0,04 |
| 22 (CART) | 0,76 | 0,82 | 0,69 |
| 22 (Logistic regression) | 0,72 | 0,77 | 0,63 |
| 22 (Random forest) | 0,88 | 0,95 | 0,63 |
| 22 (XGboost) | 0,77 | 0,86 | 0,56 |
| 24 (ADA1) |  | 0,88 | 0,89 |
| 24 (ADA2) |  | 0,92 | 0,95 |
| 24 (GDB) |  | 0,96 | 0,95 |
| 25 | 0,84 |  |  |
| 26 |  |  |  |
| 27 | 0,93 | 0,87 | 0,91 |
| 28 |  | 0,87 |  |
| 29 (model Rio Grande do Sul) |  | 0,98 | 0,22 |
| 29 (model mato grosso) |  | 0,93 | 0,81 |
| 30 (SVM) | 0,864 | 0,86 | 0,60 |
| 30 (car) | 0,864 | 0,71 | 0,65 |
| 30 (PERCEPTRONS) | 0,864 | 1,00 | 0,80 |
| 30 (MLR) | 0,864 | 0,79 | 0,85 |
| 30 (SVM LINEAR) | 0,864 | 0,86 | 0,75 |
| 30 (SVM RBF) | 0,864 | 0,86 | 0,75 |
| 31 |  | 0,78 | 0,80 |
| 32 CART | 0,702 | 0,71 | 0,65 |
| 32 Multilayer perceptron network | 0,918 | 1,00 | 0,80 |
| 32 Multivariate logistic regression | 0,782 | 0,79 | 0,85 |
| 32 SVM linea | 0,796 | 0,86 | 0,75 |
| 32 SVM polynomial | 0,782 | 0,86 | 0,60 |
| 32 SVM RBF | 0,793 | 0,86 | 0,75 |
